# Culturally Considerate Trauma-Focused Post-Traumatic Stress Disorder Treatment in Latine/x Populations: A Scoping Review

**DOI:** 10.3390/healthcare13050469

**Published:** 2025-02-21

**Authors:** Alejandra Kukuli Delgado, Ryan Holliday, Shira Maguen, Nicholas Holder

**Affiliations:** 1 San Francisco VA Health Care System, 4150 Clement St, San Francisco, CA 94121, USA; shira.maguen@va.gov (S.M.); nicholas.davis.holder@gmail.com (N.H.); 2 Department of Psychiatry and Behavioral Sciences, University of California-San Francisco, San Francisco, CA 94107, USA; 3 Rocky Mountain Mental Illness Research, Education and Clinical Center for Suicide Prevention, Aurora, CO 80045, USA; ryan.holliday@va.gov; 4 Department of Psychiatry, University of Colorado Anschutz Medical Campus, Aurora, CO 80045, USA; 5 Center for Data to Discovery and Delivery Innovation (3DI), 4150 Clement St, San Francisco, CA 94121, USA

**Keywords:** PTSD, Latine/x, trauma-focused psychotherapy, culturally considerate treatment, scoping review

## Abstract

**Background/Objectives**: While theoretical articles describing cultural considerations for post-traumatic stress disorder (PTSD) treatment in Latine/x populations exist, empirical trials are less common. The present study aimed to review the existing literature for empirically tested, trauma-focused PTSD treatments among Latine/x samples to describe treatment outcomes, cultural considerations, and gaps in the existing literature. **Methods:** For this scoping review, we identified studies that met inclusion criteria as culturally considerate, trauma-focused PTSD treatments reporting symptom outcomes in Latine/x adults. We searched databases (*n* = 7) and collected 2176 unique records. Six reports met inclusion criteria. Studies enrolled 176 participants into five trauma-focused interventions (e.g., prolonged exposure therapy) and three comparison treatments (e.g., muscle relaxation). **Results:** Cultural considerations varied from Spanish translation to measuring *nervios* to the application of cultural values (e.g., *familismo, personalismo*) in session. Although culturally considerate treatment was associated with PTSD symptom reduction, none of the studies included unmodified trauma-focused treatment as a comparison group. Literature gaps included heterogeneity in methods, treatments, and cultural considerations, which impede the synthesis of current literature and translation from theory to practice. **Conclusions:** Inferences about the incremental benefits of cultural considerations are limited due to the absence of comparison to unmodified PTSD treatment. By identifying the existing literature gaps, this study aims to support optimal and culturally appropriate treatment for PTSD in Latine/x populations.

## 1. Introduction

While Latine/x individuals make up 17% of the current U.S. population, this rapidly growing group is estimated to reflect 29% of the population by 2060 [1]. Given the fast growth of this population, it is important to be prepared to address mental health needs of Latine/x individuals. The estimated lifetime prevalence of PTSD in the U.S. population is 6% [2], and the current data suggest that Latine/x populations are among the sociodemographic groups with the highest rates of PTSD [3]. The existing literature indicates that these differences in PTSD among Latine/x individuals may be influenced by social disadvantage, peri-traumatic response, expressive style, acculturation, cultural values (i.e., familism, fatalism), culturally sanctioned practices (e.g., Hispanic Stoicism), and coping strategies [4,5]. Considering cultural factors in mental health treatment is critical to enhance effectiveness and accessibility in interventions across diverse populations as well as reducing mental health disparities [6,7].

PTSD treatment research consistently recommends evidence-based, trauma-focused, cognitive behavioral therapy to treat PTSD [8,9,10]. Unfortunately, Latine/x individuals may not be well represented in the large body of evidence supporting trauma-focused PTSD treatment [11]. The research that has examined cultural factors impacting PTSD onset and course in Latine/x individuals has also encouraged the use of Latine/x cultural theoretical studies to adjust treatment [12,13]. Indeed, this is seen through studies that have developed modifications to trauma-focused treatment with the intention of making them culturally considerate [14]. Importantly, the consideration of culture in PTSD treatment has shown to be essential in ensuring that interventions are effective and align with patients across diverse backgrounds [15].

*Culturally considerate psychotherapy* acknowledges culture and its role in treatment while generating actionable steps toward culturally informed interactions and adjustments to treatment (e.g., culturally sensitive approaches, formal cultural adaptations, etc.; [16]). Two important aspects of culturally considerate psychotherapy are cultural sensitivity and cultural adaptations. *Cultural sensitivity* emphasizes professionals’ practice awareness of self and others, and the employment of one’s knowledge, consideration, understanding, and respect to tailor and facilitate effective interactions and interventions [16]. *Cultural adaptations* are systematic modifications of the treatment protocol to consider language, culture, or context consistent with a patient’s cultural patterns, meanings, and values [17].

While developing culturally considerate treatment represents an important first step to ensuring high-quality PTSD treatment for Latine/x individuals, empirically testing these interventions is needed to confirm the appropriate transition of theory to practice [18]. Considering the growing Latine/x population, strong evidence for efficacy of trauma-focused PTSD treatment, and the potential for unique cultural needs for Latine/x individuals, the present scoping study sought to review the existing literature for empirically tested, trauma-focused PTSD treatments among Latine/x samples to describe treatment outcomes, cultural considerations, and gaps in the existing literature.

## 2. Method

The methods and procedures for this review were informed by the Arksey and O’Malley (2005; [19]) methodological framework on scoping reviews. The present review followed the five stages (identifying research question; identifying relevant studies; study selection; charting data; and collating, summarizing, and reporting results) outlined in this framework to describe research in the topic area and identify gaps in the extant literature. The present review complies with the Preferred Reporting Items for Systematic reviews and Meta-Analyses extension for Scoping Reviews (PRISMA-ScR) guidelines (see Supplementary Figure S1; Tricco et al., 2018; [20]).

### 2.1. Search Strategy

Seven databases were queried with customized search formulas for each database, including PubMed, APA Psych Info, APA Psych Articles, CINAHL Plus, OVID, EMBASE, and Google Scholar (see Supplementary Table S2). For each database, preliminary searches were run to confirm best fit search formulas, and final terms were chosen based on output quantities (i.e., excluded formulas that were overly inclusive). *Hispanic* or *Latino/a/x/e* or *Spanish* and *post-traumatic stress disorder* were the umbrella terms used to create a representative output. To identify all studies with Latine/x participants, we utilized broader search terms (e.g., “Hispanic”) due to potential inconsistent/inaccurate use of terms in the prior literature and to assure an extensive search. Using fewer terms that were more specific resulted in fewer results compared to the selected search formulas, and adding terms did not generate substantial additional results. Searches were conducted on 20 May 2024.

### 2.2. Eligibility Criteria

Reports met inclusion criteria if (1) a trauma-focused psychotherapy was empirically tested (to ensure that there were outcomes available for adapted interventions); (2) participants had clinically meaningful symptoms of post-traumatic stress disorder as assessed by an empirically supported measure (to ensure the intervention was intended to treat PTSD); (3) individuals were ≥18 years old; (4) studies included Latine/x-only samples (to assess treatment details and cultural components that are targeted specifically for Latine/x populations); and (5) research occurred in the United States of America or its territories. Reports in English or Spanish were included. There was no restriction on the publication year. A variety of study designs were eligible for inclusion (i.e., randomized control trials, feasibility trials, pilot trials, etc.); however, case studies and secondary analyses were not included. According to Arksey and O’Malley (2005; [19]), we included gray literature (i.e., dissertations) that met eligibility criteria.

### 2.3. Data Collection Process and Data Items

**Figure 1:**
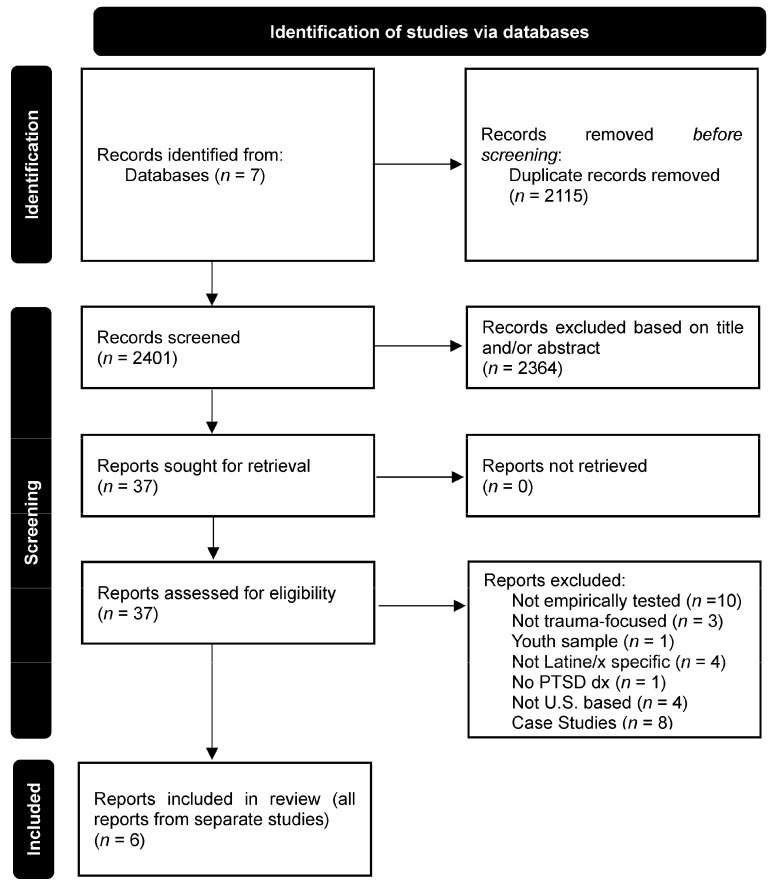
PRISMA 2020 flow diagram for scoping review.

The primary search of the seven databases yielded 4516 records, with 2401 unique records identified after deduplication. Records were assessed for inclusion criteria first by title/abstract, then by full text (see Figure 1; Page et al., 2021; [21]). Reports then underwent full-text review for inclusion (*n* = 37) by two authors (AD and NH). Interrater reliability was moderate (κ = 0.77, 94.6% percent agreement; [22]). Authors discussed discrepancies and came to a consensus regarding the inclusion of reports. Reference lists of the six included reports were reviewed to identify reports missed by the search; however, no additional reports were identified.

### 2.4. Data Charting

Data from eligible studies were charted using a standardized data abstraction tool designed for this study. The tool captured the relevant information on study design, treatment type, comparison treatment condition, study location, sample size, inclusion criteria, sample characteristics, measure for establishing clinically meaningful PTSD symptoms, PTSD treatment outcome measures, other outcome measures, the cultural components of treatment, cultural adaptation frameworks used, PTSD symptom results, treatment completion, and other outcome results (see Table 1 and Table 2).

### 2.5. Collating, Summarizing, and Reporting Results

After data charting, we summarized all of the study information to provide a basic overview of the studies included (i.e., study design, treatment type, comparison treatment condition, study location, sample size, inclusion criteria, sample characteristics, measure for establishing clinically meaningful PTSD symptoms, PTSD treatment outcome measures, and other outcome measures). Next, we summarized the cultural considerations (i.e., the cultural components of treatment, cultural adaptation frameworks used, etc.) and treatment outcomes (i.e., PTSD symptom results, treatment completion, and other outcome results) associated with the studies.

## 3. Results

### 3.1. Study Descriptions

We identified six studies that tested trauma-focused PTSD treatment for Latine/x samples (see Table 1 and Table 2). The treatments tested were written exposure therapy (WET; [29]), prolonged exposure therapy (PE; 2 studies; [30]), cognitive behavioral therapy (CBT) for PTSD and somatization [25], culturally adapted (CA)-CBT [27,31], and cognitive processing therapy (CPT; [32]). See Supplementary Table S2 for treatment descriptions. PTSD diagnosis was established most commonly through the clinically administered PTSD Scale (CAPS for DSM-IV or DSM-5), with one study using the Structured Clinical Interview for DSM-IV (SCID). Some studies also determined clinically meaningful PTSD symptoms through self-report measures, including the PTSD Checklist (PCL for DSM-IV or DSM-5) and PTSD Symptom Scale. Five studies were pilot trials, in which three studies randomized participants to a comparison treatment condition (i.e., Applied Relaxation [AR], Applied Muse Relaxation [AMR], and Usual Care [UC]). The sixth study was a pilot trial that switched to a qualitative study design due to high rates of attrition. Studies were published between 2010 and 2022 and had sample sizes ranging from 11 to 98 participants. Studies were conducted across the United States and its territories: two studies took place in Puerto Rico, three studies took place on the east coast, and one in the midwest. One study had an all-male sample, two studies recruited an all-women sample, and three studies recruited participants of all genders. All studies included Latine/x-only participants, but country of origin largely varied. Approximately 72.3% of participants across studies were born in Puerto Rico, 13.6% were born in Mexico, 5.6% were born in the Dominican Republic, and 8.5% were born in other countries in Central and/or South America. Percentages are approximate, as two studies did not report country of origin specific to participants that started treatment.

**Table 2:** Study results.

	Study	Cultural Components of Treatment	Cultural Adaptation Frameworks	PTSD Results	Treatment Completion	Other Outcome Results
Peer-Reviewed Manuscripts	Andrews et al., 2022 [23]	• Forward and back protocol translation • Flexibility in location and scheduling • Spanish-speaking therapists	None	• Significant pre- to post-treatment reduction in PCL-IV (*d* = 1.37; ITT). • A total of 75% (12/16) of participants had meaningful PTSD symptom reduction (≥13 points on PCL-IV).	• Overall, 75% (12/16) completed treatment (all five sessions).	• Depression: significant pre- to post-treatment reduction in PHQ-9 (*d* = 1.01; ITT).
	Vera et al., 2021 [24]	• Treatment translation • Used idioms and culturally relevant examples • Used culturally relevant examples of traumatic experiences • Spanish-speaking therapists • Engagement session with option to bring a relative	Comprehensive stage approach	• Significant pre- to post-treatment reduction in CAPS-5 for PE (*d* = 1.29; ITT) and AR (*d* = 1.38; ITT). • No significant difference in CAPS-5 change between treatments. •Significant pre-to post treatment reduction in PCL-5 for PE and AR (*p* < 0.001). • PCL-5: No significant difference in PCL—5 changes between treatments.	• Overall, 69.4% (34/49) completed PE (12–15 sessions). • Overall, 71.4% (35/49) completed AR (12–15 sessions). • No significant difference in treatment completion between treatments.	• Depression: significant pre- to post-treatment reduction in PHQ-9 for PE (*d* = 0.94; ITT) and AR (*d* = 1.10; ITT). • Depression: no significant difference in PHQ-9 change between treatments. • Anxiety: significant pre- to post- treatment reduction in STAI-S for PE (*d* = 1.02; ITT) and AR (*d* = 1.13; ITT). • Anxiety: no significant difference in STAI-S change between treatments.
	Perez Benitez et al., 2013 [25]	• Considered cultural beliefs and terms about trauma and symptoms • Addressed the usage of therapy as “desahogo” for Latin Americans • Providers had familiarity with historical and legal/immigration issues. • Therapist and supervisor were fully bilingual and bicultural allowing participants to alternate between Spanish and English in sessions.	None	• No significant pre- to post- treatment reduction in CAPS-IV. • No significant pre- to post- treatment reduction in CAPS-IV re-experiencing, avoidance, or hypervigilance. • Overall, 50% (4/8) did not meet criteria for PTSD diagnosis at post-treatment.	• Overall, 90.9% (10/11) completed CBT—PTSD and somatization (10–14 sessions).	• Depression: significant pre- to post-treatment reduction in BDI (*d* = 0.53). • Household functioning: significant pre- to post-treatment improvement in LIFE-Psychosocial (*d =* 1.12). • Life satisfaction: significant pre- to post-treatment improvement in LIFE-Psychosocial (*d* = 0.74). • Somatization: 37.5% “very much improved”, 25% “much improved”, 25% “minimally improved”, and 12.5% “no change” as measured by CGI- Improvement. • Physical functioning: no significant pre- to post-treatment improvement in MOS/RAND-36. • Post-traumatic cognitions: no significant pre- to post-treatment changes were found in the PTCI.
	Vera et al., 2011 [26]	• Spanish translation with emphasis on conceptual and cultural equivalence in language • Introductory session with option to bring spouse/partner	None	• Significant pre- to post-treatment reduction in CAPS-IV for PE. • Significant pre- to post-treatment reduction in CAPS-IV for UC. • Significant quadratic group x time interaction (*p* = 0.01) and no significant linear group x time interaction (*p* = 0.07).	• Overall, 71.4% (5/7) completed PE (12–15 sessions). • Overall, 100% (7/7) completed UC (12–15 sessions). • No significant difference in treatment completion between treatments.	• N/A
	Hinton et al., 2011 [27]	• Treatment was translated. • Sociocultural adaptations (i.e., loving kindness meditation through Christian imagery; modifying catastrophic cognitions by addressing cultural syndromes; teaching emotion regulation through religious imagery; presenting key lessons with culturally appropriate analogies; and modification of fear networks through palm tree visualizations) • Addressed treatment barriers (i.e., low English skills, minimal formal education, lack of familiarity with Western psychology, prominent somatic complaints, culturally specific syndromes, idioms of distress, and understanding symptoms) • Provider fluent in Spanish	None	• Significant pre- to post-treatment reduction in CAPS-IV for CA-CBT (*d* = 2.6; ITT) and AMR (*d* = 0.8; ITT). • Significantly greater reduction in CAPS-IV in CA-CBT than AMR (*d* = 1.6; ITT).	• Overall, 100% (12/12) completed CA-CBT (14 sessions). • Overall, 100% (12/12) completed AMR (14 sessions). • No significant difference in treatment completion between treatments.	• Anxiety: significant pre- to post- treatment reduction in SC1-Anxiety Scale for CA-CBT (*d* = 1.6; ITT) and AMR (*d* = 0.5; ITT). • Anxiety: significantly greater reduction in SC1-Anxiety Scale in CA-CBT than AMR (*d* = 1.1; ITT). • Nervios: significant pre- to post- treatment reduction in Nervios Scale for CA-CBT (*d* = 1.8; ITT) and AMR (*d* = 0.4; ITT). • Nervios: significantly greater reduction in Nervios Scale in CA-CBT than AMR (*d* = 1.0; ITT). • Emotion regulation: significant pre- to post-treatment reduction in ERS for CA-CBT (*d* = 3.0; ITT) and AMR (*d* = 0.6; ITT). • Emotion regulation: significantly greater reduction in ERS in CA-CBT than AMR (*d* = 2.0; ITT).
Not Peer-Reviewed (Dissertation)	Rodriguez, 2011 [28]	•Therapy sessions in Spanish • Sessions held in a familiar location • Flexibility in scheduling • Handouts were translated into Spanish • Adaptions were made according to the education level of participants • Flexibility with in-session tasks (e.g., one-word answers, drawings)	None	• N/A—no statistical analysis due to high attrition.	• Overall, 11.1% (1/9) completed CPT (12 sessions).	• N/A—no statistical analysis due to high attrition.

Notes: BDI = Beck Depression Inventory; CGI = Clinical Global Impression; CAPS-IV/5 = Clinician-Administered PTSD Scale for DSM-IV/5; DSM-IV/5 = Diagnostic and Statistical Manual of Mental Disorders-IV/5; ERS = Emotion Regulation Scale; LIFE = Longitudinal Interval Follow-up Evaluation; MOS-RAND = Medical Outcomes Study; PCL-IV = PTSD Checklist for DSM-IV; PHQ-9 = Patient Health Questionnaire; PSS = PTSD Symptom Scale-Self Report PTCI = Post-traumatic Cognitions Inventory; SCID = Structured Clinical Interview; SCL = Symptom Checklist; STAI-S = State Trait Anxiety Inventory State Version.

### 3.2. Cultural Considerations

All studies aimed to evaluate the efficacy of trauma-focused PTSD treatment for Latine/x individuals after adjusting treatment protocols for cultural considerations (i.e., Spanish translation, addressing cultural beliefs/other cultural factors, inclusion of culturally relevant outcome measures, treatment barriers) in Latine/x cultures. Five studies completed treatment sessions in Spanish. Several of the studies targeted cultural idioms, examples, and phrases that were culturally aligned and relevant to Latine/x culture and the traumatic experiences commonly faced by Latine/x people. For example, Vera et al. (2021; [24]) gave particular attention to ensuring that language was culturally syntonic, utilizing metaphors common in Latine/x communities and treatment concepts concordant with Latine/x culture and context.

Four studies addressed cultural beliefs or cultural factors specific to Latine/x populations [24,25,26,27]. Another study highlighted provider familiarity with historic events in Latin America and study therapists used the knowledge of legal/immigration issues to establish rapport [25]. Further, two studies added a pre-treatment introductory/engagement session where participants had the option of bringing a loved one as a way to address high levels of familism and interdependence prominent in Latine/x culture [24,26]. Perez Benitez et al. (2023; [25]) acknowledged Latine/x individual’s view of therapy as “desahogo (i.e., a way to get everyday problems off their chest) through active and reflective listening during therapy sessions to create a balance between “desahogo” and CBT treatment session goals.

Four studies used measurement and integration of cultural understanding of PTSD. Hinton et al. (2011; [27]) addressed culturally bound syndromes (e.g., ataque *de nervios* or “attack of nerves”) by assuring the patients’ understanding of the physiology of these symptoms, and by including an outcome measure for culturally bound syndromes, *nervios* (“nerves” in English) and *ataque de nervios*, relevant for Latine/x populations [27]. Two studies also assessed somatic complaints, which are more frequently reported manifestations of mental health symptoms among Latine/x populations [25,27]. These studies addressed somatic complaints through psychoeducation, breathing retraining [27] and relaxation training [25]. Other studies addressed common treatment barriers experienced by many underserved communities, including Latine/x populations [23,27,28]. For example, Andrews et al. (2022; [23]) addressed treatment access, including transportation and time, by allowing flexibility in scheduling and location, and Hinton et al. (2011; [27]) addressed lack of familiarity with Western psychological concepts by using terms that are easily explained such as emotional and psychological flexibility.

Of the included studies, only Vera et al. (2021; [24]) reported the use of a theoretical framework to guide the cultural adaptation process. Specifically, this study used a “comprehensive stage model,” derived from staged approaches previously developed by Barrera et al. (2013; [33]) and Naeem et al. (2016; [34]). To guide adaptation, these stage models encourage the following: (1) information gathering, (2) producing guidelines for adaptation, (3) translation and adaption of therapy material, and (4) field testing of adapted therapy.

### 3.3. Treatment Outcomes

Of the six identified studies, five had a sufficient sample size for intended statistical analyses, representing a total of eight treatment arms. While our review focused on PTSD symptom change, most studies also included a variety of other outcomes, including rates of treatment completion and change in other mental health symptoms (e.g., depression, anxiety, etc.).

#### 3.3.1. PTSD Treatment Outcomes

Significant pre- to post-treatment PTSD symptom change was observed in seven of eight treatment arms (i.e., WET, PE (two studies), CA-CBT, AR, AMR, UC). Effect sizes were reported for five treatment arms (i.e., WET, PE, CA-CBT, AR, AMR), with interventions showing a medium to large improvement in PTSD symptoms (from *d* = 0.80 for AMR to *d* = 2.6 for CA-CBT; [27]). No significant differences in PTSD symptoms from pre- to post-treatment were observed in CBT for PTSD and somatization (*p* = 0.210). Among studies with a comparison treatment condition, two out of three showed significantly greater reduction in PTSD symptoms among trauma-focused interventions than in non-trauma-focused comparison treatment conditions. Specifically, PE outperformed UC [26] and CA-CBT outperformed AMR [27]. Statistically similar PTSD symptom change was observed for PE and AR [24]. Three studies also reported the loss of PTSD diagnosis outcomes in five treatment conditions (i.e., PE (two studies), CBT for PTSD and somatization, AR, UC). Most conditions showed high rates of loss of diagnosis, but rates were variable across treatment conditions (from 0% in UC for Vera et al. (2011; [26]) to 71.8% in PE for Vera et al. (2021; [24]). In addition, Andrews et al. (2022; [23]) reported that 100% of participants who completed all sessions of treatment (*n* = 12) no longer fell within the authors’ defined clinical range of PTSD symptoms (defined as PCL-IV < 40); however, this study included participants with a “*likely* PTSD diagnosis” (i.e., PCL-IV *≥* 45).

#### 3.3.2. Treatment Completion

Sample sizes were generally small, with enrollment ranging from 11 to 98 participants [24,25]. Rates of treatment completion were calculated based on the number of participants who started treatment that attended all intended treatment sessions. Rates of study completion were highly variable. Studies with no comparison treatment condition had the largest differences in treatment completion—from 11% (1/9) of participants that started CPT [28] to 90.1% (10/11) of participants that started CBT for PTSD and somatization [25]. Study trials with a comparison treatment condition tended to have higher rates of treatment completion—from 69.4% (PE; [24]) to 100% (CA-CBT, AMR, UC; [26,27]). No significant differences in treatment completion were observed between trauma-focused and comparison treatment conditions in any study.

#### 3.3.3. Mental Health Symptoms

Studies most commonly included depression and anxiety as additional mental health outcomes; however, a wide range of mental health outcomes were assessed. A significant decrease in depression symptoms from pre- to post-treatment was reported in five treatment arms (WET, PE, AR, CBT for PTSD and somatization). Effect sizes were reported for three of the treatment arms (WET, PE, AR) with interventions showing a medium-to-large reduction in depression symptoms (from *d* = 0.94 for PE to *d* = 1.96 for WET). The only study with comparison treatment conditions which reported depression symptoms demonstrated a similar reduction in both treatment arms (PE and AR). A significant decrease in anxiety symptoms was reported in four treatment arms for two studies (i.e., PE, AR, CA-CBT, AMR). Results indicated a medium-to-large reduction in anxiety symptoms across treatment conditions (from *d* = 0.50 for AMR to *d* = 1.6 for CA-CBT). Statistically similar anxiety symptom reductions were observed for PE and AR, while CA-CBT had greater anxiety symptom reductions than AMR (*d* =1.1; [27]). Perez Benitez et al. (2013; [25]) reported on a variety of outcome measures (e.g., somatization, physical functioning, psychosocial functioning), and most showed improvement following treatment with medium-to-large effect sizes. Hinton et al. (2011; [27]) found greater improvement in CA-CBT than AMR for culturally relevant symptoms of *nervios* (between-group effect size *d* = 1.0) and emotional regulation (between-group effect size *d* = 2.0).

## 4. Discussion

This scoping study aimed to review the existing literature for empirically tested, trauma-focused PTSD treatments among Latine/x samples to identify cultural considerations, treatment outcomes, and gaps in the literature. Six reports were identified to have met inclusion criteria. A majority of treatment arms demonstrated medium-to-large pre- to post-treatment reductions in PTSD symptoms, consistent with the larger literature on trauma-focused PTSD treatment [35,36,37,38]. Studies typically showed high treatment completion rates compared to other trauma-focused PTSD treatment trials [39]. Although most of the included studies tested different trauma-focused treatments and had small sample sizes, these results provide the initial support for the efficacy of trauma-focused treatments in Latine/x populations. The state of the current literature makes it difficult to determine whether trauma-focused treatments for PTSD are superior to non-trauma-focused treatments and the degree of benefit of cultural considerations for Latine/x populations due to a lack of studies comparing adapted to non-adapted trauma-focused treatments.

The six studies in this review incorporated a variety of cultural considerations. Importantly, only one of the studies used a cultural adaptation framework to guide their adaptation process. The considerations were clustered into three overarching cultural components, i.e., the use of Spanish language, the incorporation of cultural values, and addressing culturally relevant psychosomatic experiences. These components were generally consistent with components found in the previous literature on cultural considerations and cultural adaptations [15,40]. Though not exclusive to Latine/x populations, the importance of addressing treatment barriers was considered in most studies included in this review.

### 4.1. Spanish Language

While only five studies reported translating treatment to Spanish, all identified study trials utilized Spanish language. One common use of Spanish in treatment was through the infusion of idioms and metaphors that are common in Latine/x cultures; however, most reports did not provide examples of the idioms or metaphors that were used. Another study allowed for the interchangeable use of Spanish and English (commonly referred to as “Spanglish”) in treatment sessions, though the frequency of Spanglish use was not reported. Studies also included bilingual therapists, supervisors, and other study team members. Incorporating Spanish language in treatment is widely recommended by cultural adaptation frameworks [41,42,43], and translation is commonly observed in culturally adapted treatments [15,44,45].

Therapists, supervisors, and other study team members in the identified studies were often Latine/x or bicultural themselves. Cultural adaptation frameworks [43,46] recommend that clinicians acknowledge and discuss cultural similarities and differences in therapy to enhance cultural sensitivity of treatment [41,43]. Further, the previous literature has explored the racial/ethnic matching in the therapy dyad and the assumptions of the presence of cultural congruency based on this matching [41]. However, it is important to highlight that Latine/x populations are not a monolithic cultural group. While Latine/x individuals do share a common language amongst other historical and sociocultural factors and have been historically classified into one group, they are an extensively heterogenous population that concurrently differ in migration narratives, indigenous influences, languages/dialects, customs, political and historical events, and other cultural factors. Together all these variables produce pronounced differences and impede the assumption of cultural congruency in the therapy dyad explicitly based on one commonality (i.e., language). Even though all studies in our review included Spanish-speaking (often bicultural) therapists, the level of cultural congruency present between the therapist and patient or its potential impact on treatment were neither measured nor reported.

### 4.2. Cultural Values

All studies acknowledged cultural values through a variety of approaches. A cultural value known as familism or *familismo*, commonly found in Latine/x individuals [47], was considered in some reviewed studies. *Familismo* is defined as an individuals’ strong identification with and attachment to (and prioritization of) their nuclear and extended family [48]. In two studies, pre-treatment engagement sessions were used to leverage *familismo* and Latine/x individual’s increased inclinations toward interdependence by providing the opportunity to bring a loved one to learn about treatment. This involvement of loved ones in trauma-focused PTSD treatment has been identified previously as an approach to boost treatment initiation and outcomes where veterans who reported the encouragement from close loved ones were twice as likely to complete treatment compared to veterans who reported their loved ones had not given them the encouragement [49].

Another cultural value considered in the reviewed studies was *personalismo*. *Personalismo* is described as a complimentary and essential value to *familismo* [50]. Most Latine/x populations value the uniqueness of inner qualities that constitute dignity of self and others, self-worth, and showing and receiving proper respect [51]. Others define *personalismo* as a style of communication in which Latine/x individuals tend to prefer personal over impersonal interactions [51]. In congruence with the previous literature, one included study explicitly addressed *personalismo* in therapy sessions by expressing genuine interest in patients’ everyday problems [25]. This was later emphasized as an important component in treatment by participants in a post-treatment focus group.

### 4.3. Culturally Relevant Psychosomatic Experiences

Some studies considered culturally bound syndromes (i.e., nervios, *ataque de nervios*) in treatment sessions and in outcome measures. *Nervios* represent complex physical, emotional, and social experiences that occur simultaneously on multiple levels and can be considered a trait of an individual or a state that an individual is experiencing. *Ataque de nervios* is a specific form of *nervios*—defined as acute, intense episodes which occur as the result of a major stressful event, particularly in the family sphere [52]. One study considered both *nervios* and *ataque de nervios* as part of their cultural adaption of treatment [27]. Hinton et al. (2011; [27]) addressed *nervios* in three ways—through psychoeducation about *nervios* in sessions, through modifying catastrophic cognitions about nervios (i.e., concerns that somatic or mental symptoms indicate a dangerous disorder of nervios), and measuring change in *nervios* using the Nervios Scale. Perez Benitez et al. (2013; [25]) addressed somatization (i.e., medically unexplained physical symptoms [MUPSs]) concurrently with PTSD due to a high rate of comorbidity between PTSD and somatization in Latine/x populations. Interestingly, these studies seem to address *nervios* and somatization differently. While Hinton et al. (2011; [27]) tended to treat *nervios* and somatization as a culturally specific expression of PTSD, Perez-Benitez et al., (2013; [25]) addressed somatization as a comorbid condition requiring additional, specific intervention components. While acknowledging culturally bound syndromes and culturally specific manifestations of distress may be important for ensuring culturally considerate care, the theoretical understanding of the overlap between these symptoms/disorders and the target disorder are important for optimal adaptation.

### 4.4. Treatment Barriers

Cultural adaptations work to reduce disparities in treatment use amongst populations that have underutilized mental health care despite having high mental health care needs. All studies reviewed in the current scoping study acknowledged or addressed treatment barriers related to disparities in care. One study acknowledged treatment barriers through qualitative interviews, but the barriers identified were not necessarily addressed in the treatment protocol [23]. Adjustments to treatment typically addressed more generalized (vs. Latine/x-specific) barriers (e.g., appointment flexibility, setting of appointments). Previous literature has explained the different types of treatment barriers that exist in Latine/x communities, including socioeconomic factors (e.g., lower income, no health insurance), cultural variables (e.g., perception of mental illness), and psychotherapeutic challenges (e.g., lack of consensus in cultural/ethnic matching in therapy dyad; [53]). Attention to treatment barriers throughout studies highlights the importance of addressing systemic sociocultural and structural barriers in health care to provide culturally considerate care.

### 4.5. Overarching Gaps in Research

Studies were not representative of the Latine/x cultural groups present in the U.S. (i.e., small sample sizes, one-gender studies, disproportionately representative of Puerto Rican participants), which limited generalizability and added to heterogeneity. Further, there was inconsistency between included studies regarding how to adapt treatment, what adaptations were necessary, and which treatments to test or adapt. Most studies in this scoping review did not report on the use of established cultural adaptation frameworks/models to guide treatment adjustment. Moreover, studies did not provide rationale behind the prioritization of the included cultural components over other potentially relevant cultural components. For example, *machismo* and its counterpart *marianismo* are deeply interwoven in Latine/x culture, both of which impact Latine/x individual’s interaction with mental health; however, no study specifically addressed these values. Importantly, no study tested treatment adaptations against unmodified treatment (or dismantled components). The disconnect between formative theoretical work or adaptation frameworks and empirical testing of treatment impedes advancement in culturally considerate research for PTSD treatment among Latine/x populations.

### 4.6. Limitations and Future Directions

The implications of the current scoping review should be considered in the context of its limitations. While the scoping review highlights important gaps in the existing research that necessitate further attention, these same limitations impact the ability to synthesize, generalize, and extrapolate study results. Strict inclusion/exclusion criteria ensured that we accurately studied a specific question of interest (i.e., research on empirically tested trauma-focused psychotherapy for PTSD among Latine/x adults in the U.S. or its territories). More relaxed inclusion/exclusion criteria may have identified other studies that are related to the question of interest, but do not directly address the research question of interest (e.g., reports describing culturally adapted treatments; studies of treatments designed within Latine/x countries; interventions for children; interventions for distress, etc.).

Future work should aim to expand the existing rigorous study trials with larger sample sizes to increase confidence of study results and include more representative samples (e.g., inclusion of various Latine/x subcultural groups) to allow generalizability of study results. Furthermore, future intervention adaptations would benefit from incorporating theoretical frameworks/models to support selection of specific cultural considerations (e.g., cultural components, terms, classifications) to establish a connection between theory and practice. To support the latter, future trials should consider reporting on the cultural adaptation frameworks/models that were utilized to guide process of adaptation. As previously mentioned, treatment barriers and cultural considerate treatment are intertwined when discussing ways to support the reduction in mental health disparities. Consistent with this, studies should continue to identify and address treatment barriers, particularly those specific to Latine/x populations.

Finally, studies would benefit from comparing culturally adapted trauma-focused treatment to non-culturally adapted trauma-focused treatment to further understand the advantages and level of necessity for culturally adapted trauma-focused treatment. To facilitate effective implementation of treatment and ensure the widest range of access to effective care, it is important for future research to identify the components of treatment that must be adapted for optimal outcomes vs. aspects where cultural sensitivity (without explicit adaptation) is appropriate. These recommended future directions would contribute to closing the gap between theory and practice, while advancing knowledge about delivering effective, consistent, and scalable PTSD treatment to Latine/x individuals. Despite these limitations, this scoping review can serve as a guide for researchers and policy advocates to continue to advance culturally considerate PTSD treatment for Latine/x populations.

### 4.7. Clinical Impact Statement

Our manuscript provides the initial support for the efficacy of trauma-focused treatments in Latine/x populations and informs the public on the cultural considerations being used in PTSD treatment for Latine/x individuals. This manuscript presents the current state of the science in translating theory to practice and informing policy regarding next steps in research and clinical practice within Latine/x populations.

## 5. Conclusions

This scoping study found that most of the study trials’ treatment arms demonstrated significant PTSD symptom reduction from pre- to-post-treatment, had high rates of loss of diagnosis, and had high retention rates providing preliminary support for the efficacy of the reviewed treatments. When adjusting treatment for Latine/x populations, three overarching cultural components were considered, i.e., (1) translation to Spanish language; (2) incorporation of cultural values (e.g., *familismo, personalismo*); and (3) consideration of culturally relevant psychosomatic experiences (e.g., nervios, *ataque de nervios*). The gaps found in the literature emphasized discontinuity between theory and reviewed trials. The suggested future directions for this area of research include (1) the explicit use of cultural adaptation frameworks/models when adapting treatments (e.g., identifying and describing the cultural adaptation model used to adapt a treatment; [54]); (2) the replication of existing trials with more rigorous methodology (e.g., larger samples, randomized designs, active comparison conditions); and (3) the careful evaluation of how much adaptation is needed to achieve optimal outcomes.

## Figures and Tables

**Table 1:** Study characteristics.

	Study	Study Design	Trauma- Focused Psychotherapy	Comparison Condition	Study Location	*n*	Inclusion Criteria	Sample Characteristics	Measure for Establishing Clinically Meaningful PTSD Symptoms	PTSD Outcome Measures	Other Outcome Measures
Peer-Reviewed Manuscripts	Andrews et al., 2022 [23]	Open Pilot Trial	Written exposure therapy	None	Nebraska	16	• *Likely* PTSD diagnosis	• Born outside contiguous U.S. • Primary language: Spanish • All genders	• PCL-IV	• PCL-IV	• Depression (PHQ-9)
	Vera et al., 2021 [24]	Randomized controlled trial	Prolonged exposure	Applied relaxation	Puerto Rico	98	• Age 18–64 • PTSD diagnosis • Spanish-speaking	• All genders	• CAPS-5	• CAPS-5 • PCL-5	• Depression (PHQ-9) • Anxiety (STAI-S)
	Perez Benitez et al., 2013 [25]	Open pilot trial	Cognitive behavioral therapy for PTSD and somatization	None	South Florida	11	• Age 18–65 • PTSD diagnosis • Abridged somatization diagnosis (medically unexplained physical symptoms) • English- and/or Spanish-speaking • Self-identified as Latina/o	• Born outside contiguous U.S. • All genders (87.5% completers were women)	• CAPS-IV (Spanish or English)	• CAPS-IV	• Depression (BDI) • Somatization (CGI-Improvement) • Physical functioning (MOS/RAND-36) • Psychosocial functioning (LIFE- Psychosocial) • Post-traumatic Cognition (PTCI)
	Vera et al., 2011 [26]	Pilot trial	Prolonged exposure	Usual care	Puerto Rico	14	• Age 18–65 • PTSD diagnosis • Spanish-speaking	• All men sample	• CAPS-IV	• CAPS-IV	• N/A
	Hinton et al., 2011 [27]	Pilot trial	Culturally adapted cognitive behavioral therapy	Applied Muscle Relaxation	Massachusetts	24	• PTSD diagnosis (treatment resistant) • Spanish-speaking (preferred language) • Self-identified Latina	• Caribbean sample (Puerto Rico and Dominican Republic) • All women sample	• SCID-DSM-IV (PTSD Module)	• PCL-IV	• Anxiety (SCL- Anxiety Scale) • Nervios and Ataque de Nervios (Nervios Scale) • Emotion Regulation (ERS)
Not Peer- Reviewed (Dissertation)	Rodriguez, 2011 [28]	Open pilot trial	Cognitive processing therapy	None	New York	13	• PTSD diagnosis • Depression diagnosis	• Mexican-born • All women sample	• PSS	• PSS	• Depression (BDI)

Notes: BDI = Beck Depression Inventory; CGI = Clinical Global Impression; CAPS-IV/5 = Clinician-Administered PTSD Scale for DSM-IV/5; DSM-IV/5 = Diagnostic and Statistical Manual of Mental Disorders-IV/5; ERS = Emotion Regulation Scale; LIFE = Longitudinal Interval Follow-up Evaluation; MOS-RAND = Medical Outcomes Study; PCL-IV = PTSD Checklist for DSM-IV; PHQ-9 = Patient Health Questionnaire; PSS = PTSD Symptom Scale-Self Report; PTCI = Post-traumatic Cognitions Inventory; SCID = Structured Clinical Interview; SCL = Symptom Checklist; STAI-S = State Trait Anxiety Inventory State Version.

## Data Availability

The original contributions presented in this study are included in the article/Supplementary Materials. Further inquiries can be directed to the corresponding author(s).

## Supplementary Materials

The following supporting information can be downloaded at: https://www.mdpi.com/article/10.3390/healthcare13050469/s1. Supplementary Figure S1: Preferred Reporting Items for Systematic reviews and Meta-Analyses extension for Scoping Reviews (PRISMA-ScR) Checklist. Supplementary Table S1: Final Search Formulas Supplementary. Table S2: Descriptions of Trauma-Focused Treatments.
