# Supplementary material for: Culturally Considerate Trauma-Focused Post-Traumatic Stress Disorder Treatment in Latine/x Populations: A Scoping Review
Source: Healthcare (Basel). 2025 Feb 21;13(5):469. doi: 10.3390/healthcare13050469 (PMC11898671; doi:10.3390/healthcare13050469)
Supplement: Supplementary file 1 [file healthcare-13-00469-s001.zip › healthcare-3445945-supplementary.pdf]

Supplementary Figure S1

Preferred Reporting Items for Systematic reviews and Meta-Analyses extension for Scoping Reviews (PRISMA-ScR) Checklist

SECTION	ITEM	PRISMA-ScR CHECKLIST ITEM	REPORTED ON PAGE #
**TITLE**			
Title	1	Identify the report as a scoping review.	1
**ABSTRACT**			
Structured summary	2	Provide a structured summary that includes (as applicable): background, objectives, eligibility criteria, sources of evidence, charting methods, results, and conclusions that relate to the review questions and objectives.	2
**INTRODUCTION**			
Rationale	3	Describe the rationale for the review in the context of what is already known. Explain why the review questions/objectives lend themselves to a scoping review approach.	3
Objectives	4	Provide an explicit statement of the questions and objectives being addressed with reference to their key elements (e.g., population or participants, concepts, and context) or other relevant key elements used to conceptualize the review questions and/or objectives.	4
**METHODS**			
Protocol and registration	5	Indicate whether a review protocol exists; state if and where it can be accessed (e.g., a Web address); and if available, provide registration information, including the registration number.	N/A
Eligibility criteria	6	Specify characteristics of the sources of evidence used as eligibility criteria (e.g., years considered, language, and publication status), and provide a rationale.	5
Information sources*	7	Describe all information sources in the search (e.g., databases with dates of coverage and contact with authors to identify additional sources), as well as the date the most recent search was executed.	5
Search	8	Present the full electronic search strategy for at least 1 database, including any limits used, such that it could be repeated.	5; supplementary material

SECTION	ITEM	PRISMA-ScR CHECKLIST ITEM	REPORTED ON PAGE #
Selection of sources of evidence†	9	State the process for selecting sources of evidence (i.e., screening and eligibility) included in the scoping review.	5
Data charting process‡	10	Describe the methods of charting data from the included sources of evidence (e.g., calibrated forms or forms that have been tested by the team before their use, and whether data charting was done independently or in duplicate) and any processes for obtaining and confirming data from investigators.	6
Data items	11	List and define all variables for which data were sought and any assumptions and simplifications made.	5,6
Critical appraisal of individual sources of evidence§	12	If done, provide a rationale for conducting a critical appraisal of included sources of evidence; describe the methods used and how this information was used in any data synthesis (if appropriate).	N/A
Synthesis of results	13	Describe the methods of handling and summarizing the data that were charted.	6
**RESULTS**			
Selection of sources of evidence	14	Give numbers of sources of evidence screened, assessed for eligibility, and included in the review, with reasons for exclusions at each stage, ideally using a flow diagram.	6, 28
Characteristics of sources of evidence	15	For each source of evidence, present characteristics for which data were charted and provide the citations.	6,7
Critical appraisal within sources of evidence	16	If done, present data on critical appraisal of included sources of evidence (see item 12).	N/A
Results of individual sources of evidence	17	For each included source of evidence, present the relevant data that were charted that relate to the review questions and objectives.	7-11
Synthesis of results	18	Summarize and/or present the charting results as they relate to the review questions and objectives.	25,26
**DISCUSSION**			
Summary of evidence	19	Summarize the main results (including an overview of concepts, themes, and types of evidence available), link to the review	11-16

SECTION	ITEM	PRISMA-ScR CHECKLIST ITEM	REPORTED ON PAGE #
		questions and objectives, and consider the relevance to key groups.	
Limitations	20	Discuss the limitations of the scoping review process.	16,17
Conclusions	21	Provide a general interpretation of the results with respect to the review questions and objectives, as well as potential implications and/or next steps.	18
**FUNDING**			
Funding	22	Describe sources of funding for the included sources of evidence, as well as sources of funding for the scoping review. Describe the role of the funders of the scoping review.	1

Supplementary Table S1

Final Search Formulas

Database	Search Formulas
Pubmed	(post traumatic stress disorder*[Title/Abstract] OR ptsd[Title/Abstract] OR posttraumatic stress disorder*[Title/Abstract] OR post-traumatic stress disorder*[Title/Abstract] OR stress disorder*, post-traumatic[Title/Abstract]) AND (hispanic*[Title/Abstract] OR latino*[Title/Abstract] OR latina*[Title/Abstract] OR latinx[Title/Abstract] OR latine*[Title/Abstract] OR spanish*[Title/Abstract])
APA PsychInfo	AB (post traumatic stress disorder OR ptsd OR posttraumatic stress disorder OR post-traumatic stress disorder OR stress disorder, post-traumatic) AND AB (hispanic OR latino OR latina OR latinx OR latine OR spanish)
APA Psych Articles	AB (post traumatic stress disorder OR ptsd OR posttraumatic stress disorder OR post-traumatic stress disorder OR stress disorder, post-traumatic) AND AB (hispanic OR latino OR latina OR latinx OR latine OR spanish)
CINHAHL Plus	AB (post traumatic stress disorder OR ptsd OR posttraumatic stress disorder OR post-traumatic stress disorder OR stress disorder, post-traumatic) AND AB (hispanic OR latino OR latina OR latinx OR latine OR Spanish)
OVID	((post traumatic stress disorder* or ptsd or posttraumatic stress disorder* or post-traumatic stress disorder* or stress disorder*, post-traumatic) and (hispanic* or latino* or latina* or latinx or latine or spanish)).ab.
Google Scholar	allintitle:"post traumatic stress disorder" OR "ptsd" OR "posttraumatic stress disorder" OR "post-traumatic stress disorder" OR "stress disorder, post-traumatic" AND "hispanic" OR "latino" OR "latina" OR "latinx" OR “latine” OR “spanish"
EMBASE	('post traumatic stress disorder':ab,ti OR ptsd:ab,ti OR 'posttraumatic stress disorder':ab,ti OR 'post-traumatic stress disorder':ab,ti OR 'stress disorder, posttraumatic':ab,ti) AND (hispanic:ab,ti OR latino:ab,ti OR latina:ab,ti OR latinx:ab,ti OR latine:ab;ti OR spanish:ab,ti)

Note: APA=American Psychological Association; CINAHL=Cumulative Index in Nursing and Allied Health Literature; EMBASE=excerpta medica database.

Supplementary Table S2

Descriptions of Trauma-Focused Treatments

Treatment	Description
Cognitive Behavioral Therapy (CBT) for Posttraumatic Stress Disorder (PTSD) and Somatization (Perez-Benitez et al., 2013)	CBT for PTSD and Somatization Disorder is delivered in a 10- to 14-session protocol with a modular format to maximize flexibility. The first module introduced cognitive restructuring and associated therapeutic techniques (e.g. thought records, downward arrow technique, and behavioral experiments). The second module introduced relaxation training and associated therapeutic techniques (e.g., diaphragmatic breathing and autogenic relaxation). The third module introduced activity regulation and associated techniques (e.g., activity scheduling and pacing). The last module introduced communication skills and associated therapeutic techniques (e.g., assertiveness, recognizing and expressing emotions). Sessions followed typical structure of cognitive therapy, including mood check-ins, a review of homework, agenda setting, assignment of new homework, and feedback. Throughout the modular protocol, the clinician learned about the patient's most distressing trauma, remained attentive to dysfunctional thoughts related to PTSD, used Socratic dialogue to explore thoughts, and emphasized elaboration and integration of the traumatic event through an autobiographical model.
Cognitive Processing Therapy (CPT; Resick, Monson, & Chard, 2024; Rodriguez, 2011).	CPT is delivered in a 12-session protocol of 60-min weekly individual psychotherapy sessions. In the first session, clinicians provide psychoeducation about PTSD and instruct patients to write a statement about the impact of the traumatic event. In the second session, participants read the impact statement, are taught to identify problem areas and cognitions (i.e., "stuck points") when thinking about the traumatic event, are taught to identify the relationships between thoughts and feelings and are encouraged to record events with their associated thoughts and

	feelings throughout the week (i.e., A-B-C worksheets). In session 3, A-B-C worksheets and stuck points are reviewed, and the clinician assigns the completion of a written trauma account that includes the details, thoughts, emotions, and sensory reactions of the traumatic event. In sessions 4-6, the clinician uses Socratic dialogue to explore and challenge trauma-related stuck points and introduces a series of worksheets designed to support autonomous exploration of stuck points. During sessions 7-12, patients complete worksheets to explore remaining trauma-related stuck points, particularly those related to specific core areas impacted by PTSD (i.e., safety, trust, power/control, esteem, and intimacy). In the final session, patients are asked to re-write the initial impact statement and review progress during treatment as well as plans for building on and maintaining gains.
Culturally Adapted-Cognitive Behavioral Therapy (CA-CBT; Hinton et al., 2004; Hinton et al., 2011)	CA-CBT is delivered in a 14-session protocol of 60-min weekly individual psychotherapy sessions. During CA-CBT, eight core elements are emphasized: (1) providing psychoeducation about PTSD; (2) training in muscle relaxation and diaphragmatic breathing procedures; (3) instruction on visualization and analogous rotational movements; (4) framing relaxation techniques as a form of mindfulness; (5) cognitive restructuring of fear networks; (6) conducting interoceptive exposure; (7) emotional processing; and (8) exploring headache and orthostatic panic. The CA-CBT manual has prompts where changes are made to personalize the treatment for the cultural group of the patient.
Prolonged Exposure Therapy (PE; Foa, Hembree, Rothbaum, & Rauch, 2019; Vera et al., 2021).	PE is delivered in a 12- to 15-session protocol of 90-min weekly individual psychotherapy sessions. In the first session the clinician discusses the rationale for PE, gathers information about the patient's symptoms and trauma, and teaches a breathing training exercise. In the second session, the clinician provides psychoeducation about PTSD, the

	rationale for *in vivo* exposure, and creates an in vivo hierarchy (i.e., avoided activities arranged in order of the predicted level of distress that they may cause the patient). In the third session, the clinician provides the rationale for imaginal exposure and begins imaginal exposure exercises. In the subsequent sessions, the clinician continues imaginal exposure exercises, processing of thoughts and feelings related to imaginal exposure, and discussions of *in vivo* exposure homework. The final session also includes a discussion of skills learned, progress, and plans for building on and maintaining gains.
Written Exposure Therapy (WET; Andrews et al. 2022; Sloan & Marx, 2019)	WET is delivered in a five-session protocol of 50-minute weekly individual psychotherapy sessions. In the first session, the clinician provides brief psychoeducation about PTSD linked to an exposure-based rationale for why writing about past traumatic events leads to symptom reduction (i.e., reducing avoidance). After psychoeducation, the clinician provides instructions for writing about the traumatic event. Patients then write for 30 minutes without the clinician present and provide as much clarity and detail about their sensory, cognitive, and emotional experiences at the time of the traumatic event. After the 30-minute writing period, the clinician processes the experience of writing about the traumatic event with the patient. Sessions 2-5 include a 30-minute writing period for the same traumatic event and 10 minutes for processing.
